# Limitation of life sustaining measures in neurocritical care: sex, timing, and advance directive

**DOI:** 10.1186/s40560-023-00714-y

**Published:** 2024-01-16

**Authors:** Stefan Yu Bögli, Federica Stretti, Didar Utebay, Ladina Hitz, Caroline Hertler, Giovanna Brandi

**Affiliations:** 1 Neurocritical Care Unit, Institute for Intensive Care Medicine, University Hospital Zurich, University of Zurich, Frauenklinikstrasse 26, 8091 Zurich, Switzerland; 2 Department of Neurology, Clinical Neuroscience Center, University Hospital Zurich, University of Zurich, Zurich, Switzerland; 3 Department of Radiation Oncology and Competence Center for Palliative Care, University Hospital Zurich, University of Zurich, Zurich, Switzerland

**Keywords:** Redirection of care, Palliation, Neurocritical care, Sex differences

## Abstract

**Background:**

The limitation of life sustaining treatments (LLST) causes ethical dilemmas even in patients faced with poor prognosis, which applies to many patients admitted to a Neurocritical Care Unit (NCCU). The effects of social and cultural aspects on LLST in an NCCU population remain poorly studied.

**Methods:**

All NCCU patients between 01.2018 and 08.2021 were included. Medical records were reviewed for: demographics, diagnosis, severity of disease, and outcome. Advance directives (AD) and LLST discussions were reviewed evaluating timing, degree, and reason for LLST. Social/cultural factors (nationality, language spoken, religion, marital status, relationship to/sex of legal representative) were noted. Associations between these factors and the patients’ sex, LLST timing, and presence of AD were evaluated.

**Results:**

Out of 2975 patients, 12% of men and 10.5% of women underwent LLST (p = 0.30). Women, compared to men, more commonly received withdrawal instead of withholding of life sustaining treatments (57.5 vs. 45.1%, p = 0.028) despite comparable disease severity. Women receiving LLST were older (73 ± 11.7 vs. 69 ± 14.9 years, p = 0.005) and often without a partner (43.8 vs. 25.8%, p = 0.001) compared to men. AD were associated with female sex and early LLST, but not with an increased in-hospital mortality (57.1 vs. 75.2% of patients with and without AD respectively).

**Conclusions:**

In patients receiving LLST, the presence of an AD was associated with an increase of early LLST, but not with an increased in-hospital mortality. This supports the notion that the presence of an AD is primarily an expression of the patients’ will but does not per se predestine the patient for an unfavorable outcome.

## Background

The provision of life sustaining treatments (LST) raises many ethical dilemmas. Patients admitted to a Neurocritical Care Unit (NCCU) are likely to face poor prognosis and significant morbidity with reduced quality of life [1, 2]. Patients admitted to a NCCU often lack capacity to participate in the decision-making process either due to an impaired level of consciousness or due to the alteration of brain function due to a disease. Because of the often acute nature of the illness, the prognostic uncertainty, discordant beliefs regarding outcome, and the potentially changing values of the patient in case of survival, end of life (EOL) discussions remain challenging [3–6]. In this patient cohort, the presence of an advance directive (AD) is of key importance to figure out, understand, and ultimately respect the patients’ wishes. In absence of an AD, surrogate decision makers (SDM) are invited to represent the patients’ values, beliefs, and wishes and to take decisions based on shared decision-making with the treating team [7]. Understanding the process leading to the limitation of life sustaining therapies (LLST) and EOL care is of paramount importance to provide patients with the best care.

Cultures, religions, and legislation influence the balance between ethical principles and beliefs during EOL processes [8–11]. There has been an increasing interest in the role of sex [12]. Sex and gender often determine social roles and influence the physician–patient relationship [13–15]. In non neuro-intensive care settings, female sex is associated with a higher likelihood of LLST [12]. Men are more likely to receive intensive care at the EOL, while women are more likely to state a preference for LLST [13, 15–17].

In our study, we focus on patients admitted to a NCCU in comparison to general intensive care units since factors associated with the EOL process in NCCU patients are poorly studied and due to the specific factors associated with acute brain injury as described above. While sex related differences in neurocritical care diseases have been detected [18–20], the influence of sex on LLST in neurocritical care remains unclear. Furthermore, the influence of other relevant factors such as the presence/contents of an AD or sociocultural aspects such as language spoken, nationality or the relationship to the surrogate decision makers (SDM) remain poorly studied in this patient cohort. The aim of this study was to investigate the EOL process with particular focus on sex related differences, timing of LLST, and the presence of an AD in a Swiss tertiary university NCCU.

## Methods

This retrospective, single-center cohort study was conducted in the NCCU of the University Hospital Zurich, Switzerland, a tertiary care hospital, in accordance with Good Clinical Practice guidelines, the provisions of the Declaration of Helsinki, and the national legal and regulatory requirements. The local Ethics Committee (Kantonale Ethikkommission Zürich; BASEC 2022–00270) approved this project.

### Patient population

All consecutive adult patients (≥ 18 years old) admitted to the NCCU of the University Hospital Zurich between January 2018 and August 2021 due to a neurological or neurosurgical diagnosis were included. Patients (or patients’ legal medical representatives) who refused to have their data analyzed for research projects were excluded.

The unit is a fully equipped tertiary NCCU with 12 beds treating 1200–1400 critically ill patients yearly. Around 520 patients are admitted yearly after elective neurosurgery. The patients are taken care of by an interdisciplinary team of intensivists, neurosurgeons, neurologists, and if necessary other specialty consultants. All neurological and neurosurgical diseases (incl. spontaneous/traumatic intracranial hemorrhages, ischemic stroke, infections of the nervous system, brain tumors, neuromuscular diseases, and seizure related disorders) with need for neurocritical care are admitted. No patients with COVID-19 are treated in the NCCU (they are treated irrespective of the admission diagnosis at a COVID-19 intensive care unit). The decision for admission to the NCCU is made by an intensive care bed manager (an intensive care physician). Patients who receive immediate LLST in the emergency department or resuscitation bay are not admitted to the NCCU but are extubated and admitted to the regular ward. In case of withdrawal of therapy of a patient already admitted to the NCCU, the patient is extubated. In instances in which the agonal phase extends over days the patient is discharged from the NCCU to the regular ward.

### Data collection

Demographics (age, sex), NCCU length of stay (LOS), severity of disease (sequential organ failure assessment score (SOFA) and the Simplified Acute Physiology Score II (SAPS II) assessed 24 h after admission), main diagnosis (differentiated into aneurysmal subarachnoid hemorrhage—aSAH, intracranial hemorrhage—ICH, ischemic stroke, cerebral tumor, epileptic disorders, or other neurological disease), and provision of LLST were extracted from the prospective Swiss-ICU registry (MDSi- Minimal Dataset for ICUs) and complemented by data extracted from the medical records. Details of the MDSi dataset have previously been reported [21]. We further reviewed the presence and severity of comorbidities (using the Charlson comorbidity index—CCI).

Regarding redirection of care we evaluated the following factors: timing of initial talk (early: within the first 24 h of NCCU care; late: afterwards), reason (patients wish—presence of AD), wish of patients SDM (representing the patients’ wish without written AD), medical reason (the swiss law allows for LLST based on medical reason if death or inacceptable quality of life are inevitable)—the reason for LLST is prospectively documented at discharge of the patient by the treating physician), outcome (NCCU mortality, in hospital mortality), the presence and contents of the AD (incl. documentation on cardiopulmonary resuscitation, intubation, intensive care therapy, artificial nutrition, LST). Furthermore, we extracted data on the patients nationality, the language spoken, their religion, their civil status (incl. no partner, widowed, partnership/married, divorced; and dichotomized as partner vs. no partner), the living conditions (living alone vs. with other people), their SDM (incl. next of kin separated by daughter/son, partner, sibling, parents, state-provided legal representative, or other), the sex of the SDM, the number of EOL discussions that were held, and the sex of the medical physician who was in charge of the talk deciding for LLST.

Patients receiving LLST were further categorized into:

1. Withholding of therapy: new or existing life-support therapy was not started or intensified (e.g. cardiopulmonary resuscitation, intubation and mechanical ventilation);
2. Withdrawal of therapy: active decision to stop or remove a life-sustaining treatment (e.g. mechanical ventilation, high inspiratory fraction of oxygen, infusion of catecholamines).

### Advance directive and end of life discussions

An AD is a written document that describes the patients’ wishes regarding the goal of treatment and or medical procedures in case of sudden, prolonged, or permanent loss of capacity. The most used AD document is provided both in a short (specifically answering whether a resuscitation, a treatment at an intensive care unit with or without mechanical ventilation is wished for as well as defining the SDM) as well as long version (including space for free writing and more in-depth questions regarding artificial nutrition/hydration, personal believes and hopes) by the swiss medical association (https://www.fmh.ch/dienstleistungen/recht/patientenverfuegung.cfm). An AD is legally binding in Switzerland.

In our unit, evaluation of EOL discussion is initiated by either the intensivists or the department (neurosurgery, neurology, and/or other) guiding the treatment. While rarely the case, EOL discussions can also be initiated by the next of kin. After an interdisciplinary consensus is reached (considering the patients’ prior quality of life, written (AD) or known wishes/expectations regarding their quality of life as well as disease specific prediction scores and the expertise of the treating team of physicians), the EOL discussions are primarily held by consultant intensivists. The intensivists document the discussions including expected prognosis, reasoning, presence of AD, comments made by SDM as well as the consent found at the end of a discussion. If wished for or deemed necessary, in difficult cases, the intensivist will be joined by a palliative care specialist, a neurosurgeon/neurologist, the nurse in charge, and/or pastoral care. Pastoral care is offered early on even before any EOL discussions are held for the support of the next of kin. The number of discussions primarily depends on the next of kins’ wishes. During the COVID-19 pandemic the number of next of kin at the bedside was reduced to two. However, EOL discussions were carried out outside of the unit and without a restriction.

### Statistical analysis

Statistical analysis was performed using SPSS version 29. Descriptive statistics are reported as counts/percentages, mean ± standard deviation, or as median including the interquartile range (IQR) as appropriate. All continuous data were tested for normality using Shapiro–Wilk’s test. Categorical or ordinal variables are compared with Pearson’s χ^2^ or Fisher’s exact test, continuous variables using Student’s t-tests or Mann–Whitney U tests for parametric and non-parametric data, respectively, where appropriate. For the analysis of the whole group, we performed both univariable analyses comparing sexes and a multivariable logistic regression for the prediction of LLST providing the odds ratios (OR) including the 95% confidence interval (95% CI). Bonferroni correction was applied to correct for multiple comparisons. For the analysis and comparison of patients who received LLST we again evaluated differences by sex performing a univariable analysis. Subgroup analyses were performed comparing patients with immediate vs. late LLST and patients with and without an AD.

### Data availability

The data is available upon reasonable request by the corresponding author.

## Results

A total of 2975 patients were admitted to the NCCU between 2018 and August 2021 (48.9% female). When considering the whole cohort, SAPS/SOFA values were higher in men vs. women (SAPS: 21 [12, 38] vs. 18 [9, 33], p = 0.001; SOFA: 3 [2, 6] vs. 2 [1, 5], p < 0.001 for men and women respectively). Age and frequency of LLST on the other hand did not differ between sexes (Age: 58 ± 17.5 vs. 57 ± 17.2 years, p = 0.38; LLST 182 (12.0%) vs. 153 (10.5%), p = 0.30, for men and women respectively). In the multivariable logistic regression analysis, increasing age (OR 1.040, 95% CI 1.029–1.051, p < 0.001), SOFA (OR 1.113, 95% CI 1.034–1.244, p < 0.001), and SAPS (OR 1.057, 95% CI 1.045–1.069, p < 0.001) were independently associated with LLST. Sex, however, was not an independent predictor of LLST (p = 0.092). Among patients who received LLST diagnosis were distributed as follows: 21.2% had an aSAH, 29.6% an ICH, 21.5% an ischemic stroke, 10.4% a brain tumor, 10.4% an epileptic disorder, while 6.9% had suffered from another disease.

### Differences in patients with LLST: sex

Women were on average 4 years older than men (men 69 ± 14.9 years vs. women 73 ± 11.7 years, p = 0.005). Clinical severity (i.e. SOFA and SAPS) as well as premorbid comorbidities (CCI) were comparable among sexes (p = 0.987). Women more frequently suffered from aSAH in comparison to men (13.7 vs. 30.1%, p = 0.006), while the other diagnosis were equally distributed between sexes.

The differences by sex considering the characteristics of AD and LLST within the cohort of patients that received LLST are presented in Table 1. Both sexes received LLST at a similar time-point (within 24 h vs. later). However, women more frequently received withdrawal (compared to withholding) of therapy than men (57.5 vs. 45.1% for women and men respectively, p = 0.028). Women more frequently had an AD (47.1% vs. 33.5 for women and men respectively, p = 0.014). The differences by sex considering the sociocultural factors within the cohort of patients that received LLST are presented in Table 2. Nationality, primary spoken language, religion showed no difference between sexes. Women, more frequently had no partner (43.8 vs. 25.8% for women and men respectively, p = 0.001). In the same line, women lived alone more frequently. When evaluating the SDM women were more frequently represented by their children while men were more frequently represented by a partner or a parent.

**Table 1:** LLST and AD characteristics depending on sex

	Male	Female	p
	182 (54.3)	153 (45.7)	
Timing (early vs. late)			
Late	114 (62.6)	103 (57.5)	0.422
Degree (withdraw vs. withhold)			
Withdraw	82 (45.1)	88 (57.5)	0.028
Reason			
Patients wish	54 (29.7)	49 (32.0)	0.671
SDMs wish	42 (23.1)	29 (19.0)	
Medical reason	86 (47.3)	75 (49.0)	
ICU mortality	61 (33.5)	60 (39.2)	0.305
In hospital mortality	115 (63.2)	113 (73.9)	0.045
Advance directive			
Present	61 (33.5)	72 (47.1)	0.014
Cardiopulmonary resuscitation			
Allow	8 (4.4)	8 (5.2)	1.000
Refuse	37 (20.3)	36 (23.5)	
Intubation			
Allow	10 (5.5)	12 (7.8)	0.805
Refuse	26 (14.3)	27 (17.6)	
ICU care			
Allow	8 (4.4)	12 (7.8)	0.768
Refuse	10 (5.5)	19 (12.4)	
Artificial feeding			
Allow	16 (8.8)	13 (8.5)	0.482
Refuse	21 (11.5)	25 (16.3)	
LST			
Allow	7 (3.8)	15 (9.8)	0.240
Refuse	49 (26.9)	55 (35.9)	
Number of EOL talks (median [IQR])	2 [1, 4]	2 [1, 4]	0.159
Sex physician			
Female	66 (36.3)	63 (41.2)	0.455

Data shown as n (%) unless otherwise stated. LLST Limitation of Life Sustaining Treatments, SDM surrogate decision maker, ICU Intensive Care Unit, LST Life Sustaining Treatment, EOL End of Life, IQR Inter Quartile Range

**Table 2:** Social and cultural characteristics depending on sex

	Male	Female	p
	182 (54.3)	153 (45.7)	
Nationality			
Swiss	153 (84.1)	131 (85.6)	0.761
Language			
German-Speaking	164 (90.1)	137 (89.5)	1.000
Religion			
Agnostic	82 (45.1)	69 (14.1)	0.778
Catholic	54 (29.7)	45 (29.4)	
Protestant	40 (22.0)	32 (20.9)	
Muslim	3 (1.6)	5 (3.3)	
Jewish	0 (0.0)	1 (0.7)	
Other	3 (1.6)	1 (0.7)	
Civil status			
Single	25 (13.7)	16 (10.5)	< 0.001
Widowed*	11 (6.0)	32 (20.9)	
Partnership/marriage*	135 (74.2)	86 (56.2)	
Divorced*	11 (6.0)	19 (12.5)	
No partner	47 (25.8)	67 (43.8)	0.001
Autonomy			
Dependent in daily life	37 (20.3)	23 (15.0)	0.253
Living situation			
Alone	42 (23.1)	55 (35.9)	0.011
SDM			
Daughter/son*	42 (23.1)	62 (40.6)	0.006
Spouse*	91 (50.0)	56 (36.6)	
Life partner	12 (6.6)	8 (5.2)	
Sibling	16 (8.8)	13 (8.5)	
KESB	3 (1.6)	5 (1.3)	
Parent*	11 (6.0)	2 (1.3)	
Other	7 (3.8)	9 (5.9)	
Sex SDM			
Female	151 (83.0)	52 (34.0)	< 0.001

Data shown as n (%) unless otherwise stated. SDM surrogate decision maker, KESB Kinder und Erwachsenen Schutzbehörde (child and adult protection authority). Subgroups marked with * were significant in the post-hoc subgroup analysis

### Differences in patients with LLST: timing

Early LLST occurred in 35.2% of patients. While the sexes were distributed equally (p = 0.422), patients with early LLST were older (73 ± 12.0 vs. 69 ± 14.3 years, for early vs. late LLST, p = 0.008), and the diagnosis was more frequently a cerebral malignancy (21.2 vs. 4.6% for early vs. late LLST), while patients with late LLST more frequently suffered from aSAH (12.7 vs. 25.8% for early vs. late LLST) or cerebrovascular ischemia (15.3 vs. 24.9% for early vs. late LLST). However, clinical severity of patients receiving early LLST was lower (SOFA: 5 [3, 9] vs. 9 [6.5, 10] p < 0.001, and SAPS: 43.5 [24, 57] vs. 55 [45, 63.5] p = 0.002 for early and late LLST respectively).

The differences by timing of LLST considering the characteristics of AD and LLST within the cohort of patients that received LLST are presented in Table 3. Patients receiving early LLST more frequently had an AD stating against reanimation, intubation, or intensive care. Early LLST was also more frequently based on the patients’ wish (55.9% vs. 17.1%, for early and late LLST respectively), while later LLST was more frequently based on the representatives wish (11.0 vs. 26.7%, for early and late LLST respectively) or most commonly a medical reason (33.1 vs. 56.2% for early and late LLST respectively). Early LLST was also associated with a shorter median NCCU LOS duration of 1 (1–3) vs. 5 (2–11) days for early vs. late LLST respectively (p < 0.001). The differences by timing of LLST considering the sociocultural factors within the cohort of patients that received LLST are presented in Table 4. Nationality, main spoken language as well as religion, marital status, type of living situation did not have an influence on the timing of LLST.

**Table 3:** LLST and AD characteristics depending on timing of LLST

	Early LLST	Late LLST	p
	118 (35.2)	217 (64.8)	
Degree (withdraw vs. withhold)			
Withdraw	36 (30.5)	134 (61.8)	< 0.001
Reason			
Patients wish*	66 (55.9)	37 (17.1)	< 0.001
SDMs wish*	13 (11.0)	58 (26.7)	
Medical reason*	39 (33.1)	122 (56.2)	
ICU mortality	27 (22.9)	94 (43.3)	< 0.001
In hospital mortality	48 (40.7)	180 (82.9)	< 0.001
Advance directive			
Present	64 (54.2)	69 (31.8)	< 0.001
Cardiopulmonary resuscitation			
Allow	4 (3.4)	12 (5.5)	0.013
Refuse	44 (37.3)	29 (13.4)	
Intubation			
Allow	4 (3.4)	18 (8.3)	0.001
Refuse	32 (27.1)	21 (9.7)	
ICU care			
Allow	5 (4.2)	15 (6.9)	0.020
Refuse	17 (14.4)	12 (5.5)	
Artificial feeding			
Allow	15 (12.7)	14 (6.5)	1.000
Refuse	24 (20.3)	22 (10.1)	
LST			
Allow	6 (5.1)	16 (7.4)	0.043
Refuse	53 (44.9)	51 (23.5)	
Number of EOL talks (median [IQR])	1 [0, 2]	3 [2, 5]	< 0.001
Sex physician			
Female	36 (30.5)	93 (42.9)	0.314

Data shown as n (%) unless otherwise stated. LLST Limitation of Life Sustaining Treatments, SDM surrogate decision maker, ICU Intensive Care Unit, LST Life Sustaining Treatment, EOL End of Life, IQR Inter Quartile Range. Subgroups marked with * were significant in the post-hoc subgroup analysis

**Table 4:** Social and cultural characteristics depending on timing of LLST

	Early LLST	Late LLST	p
	118 (35.2)	217 (64.8)	
Nationality			
Swiss	102 (86.4)	182 (83.9)	0.633
Language			
German-Speaking	110 (93.2)	191 (88.0)	0.184
Religion			
Agnostic	49 (41.5)	102 (47.0)	0.374
Catholic	37 (31.4)	62 (28.6)	
Protestant	29 (24.6)	43 (18.8)	
Muslim	1 (0.8)	7 (3.2)	
Jewish	1 (0.8)	0 (0.0)	
Other	1 (0.8)	3 (1.4)	
Civil status			
Single	17 (14.4)	24 (11.1)	0.430
Widowed	14 (11.9)	29 (13.4)	
Partnership/marriage	80 (67.8)	141 (65.0)	
Divorced	7 (5.9)	23 (10.6)	
No partner	38 (32.2)	76 (35.0)	0.631
Autonomy			
Dependent in daily life	28 (23.7)	32 (14.7)	0.052
Living situation			
Alone	32 (27.1)	65 (30.0)	0.614
SDM			
Daughter/son	32 (27.1)	72 (33.2)	0.475
Spouse	62 (52.5)	85 (39.2)	
Life partner	7 (5.9)	13 (6.0)	
Sibling	8 (6.8)	21 (9.7)	
KESB	1 (0.8)	4 (1.8)	
Parent	4 (3.4)	9 (4.1)	
Other	4 (3.4)	12 (5.5)	
Sex SDM			
Female	77 (65.3)	126 (58.1)	0.242

Data shown as n (%) unless otherwise stated. LLST Limitation of Life Sustaining Treatments, SDM surrogate decision maker, KESB Kinder und Erwachsenen Schutzbehörde (child and adult protection authority)

### Differences in patients with LLST: advance directive

39.7% of patients receiving LLST had a written advance directive. These were more frequently female (33.5 vs. 47.1%, for men and women having an AD respectively, p = 0.014) and older (67 ± 14.8 vs. 75 ± 10.3 years, for patients without and with AD respectively, p < 0.001). Median NCCU LOS in patients with AD was shorter (2 (1–6) vs. 3 (1–9) days, for patients without and with AD respectively, p = 0.039).

The differences by presence of AD considering the characteristics of LLST within the cohort of patients that received LLST are presented in Table 5. Even though patients with an AD were less severely ill (SOFA: 9 [6, 10] vs. 7 [4, 9] p < 0.001; SAPS 52.5 [43, 63] vs. 47 [30, 58.5] p = 0.001, for patients without and with AD respectively) timing of LLST was earlier (late LLST in 73.3% of patients without AD and 51.9% of patients with AD, p < 0.001) and LLST was more frequently based on the patients’ wishes. The presence of an AD was not associated to an increased but decreased in-hospital mortality (75.2 vs. 57.1% for patients without and with AD respectively, p = 0.001). The differences by presence of AD considering the sociocultural factors within the cohort of patients that received LLST are presented in Table 6. Patients with an AD were more frequently Swiss (79.7 vs. 92.5% for patients without and with AD respectively, p = 0.002) and more frequently German speaking (84.7 vs. 97.7% for patients without and with AD respectively, p < 0.001). Interestingly neither religion, nor living situation or civil status were different between patients with/without AD.

**Table 5:** LLST characteristics depending on presence of an AD

	No AD	AD	p
	202 (60.3)	133 (39.7)	
Timing (early vs. late)			
Late	148 (73.3)	69 (51.9)	< 0.001
Degree (withdraw vs. withhold)			
Withdraw	121 (59.9)	49 (36.8)	< 0.001
Reason			
Patients wish*	32 (15.8)	71 (53.4)	< 0.001
SDMs wish*	53 (26.2)	18 (13.5)	
Medical reason*	117 (57.9)	44 (33.1)	
ICU mortality	86 (42.6)	35 (26.3)	0.003
In hospital mortality	152 (75.2)	76 (57.1)	0.001
Number of EOL talks (median [IQR])	2 [1, 5]	2 [0.5, 3]	0.001
Sex physician			
Female	83 (41.1)	46 (34.6)	0.569

Data shown as n (%) unless otherwise stated. AD advance directive, LLST Limitation of Life Sustaining Treatments, ICU Intensive Care Unit, EOL End of Life, IQR Inter Quartile Range. Subgroups marked with * were significant in the post-hoc subgroup analysis

**Table 6:** Social and cultural characteristics depending on the presence of an AD

	No AD	AD	p
	202 (60.3)	133 (39.7)	
Nationality			
Swiss	161 (79.7)	123 (92.5)	0.002
Language			
German-Speaking	171 (84.7)	130 (97.7)	< 0.001
Religion			
Agnostic	94 (46.5)	57 (42.9)	0.653
Catholic	58 (28.7)	41 (30.8)	
Protestant	41 (20.3)	31 (23.3)	
Muslim	6 (3.0)	2 (1.5)	
Jewish	0 (0.0)	1 (0.8)	
Other	3 (1.5)	1 (0.8)	
Civil status			
Single	26 (12.9)	15 (11.3)	0.886
Widowed	24 (11.9)	19 (14.3)	
Partnership/marriage	133 (65.8)	88 (66.3)	
Divorced	19 (9.4)	11 (8.3)	
No partner	69 (34.2)	45 (33.8)	0.951
Autonomy			
Dependent in daily life	30 (14.9)	30 (22.6)	0.081
Living situation			
Alone	57 (28.2)	40 (30.1)	0.712
SDM			
Daughter/son	54 (26.7)	50 (37.6)	0.305
Spouse	90 (44.6)	57 (42.9)	
Life partner	13 (6.4)	7 (5.3)	
Sibling	19 (9.4)	10 (7.5)	
KESB	4 (2.0)	1 (0.8)	
Parent	10 (5.0)	2 (1.5)	
Other	0 (0.0)	6 (4.5)	
Sex SDM			
Female	123 (60.9)	80 (60.2)	0.892

Data shown as n (%) unless otherwise stated. AD advance directive, SDM surrogate decision maker, KESB Kinder und Erwachsenen Schutzbehörde (child and adult protection authority)

## Discussion

Women more frequently received withdrawal instead of withholding of LST in comparison to men despite comparable severity of disease and despite similar overall frequencies of LLST. Older age and medical comorbidities have been associated with LLST [12]. While the females in our cohort were on average 4 years older, the frequency and severity of comorbidities was comparable. Men admitted to an intensive care unit are less likely to have a limitation of care order in place [22]. In our cohort 40% of patients had an AD including almost half of all females and only close to a third of all males. Our results reveal some important social and cultural aspects that could have played a role in women deciding to write an AD: women were frequently living alone and without a partner, while men were more frequently married. This reflects the demographic portrait of Switzerland, in which women have a longer life expectancy and are less prone to find a new partner after a divorce or bereavement [23]. The development of an AD also helps alleviate stress felt by relatives who are burdened by taking EOL decisions as SDM [24]. Women were almost twice as likely to have their son/daughter as their SDM and potentially aimed at lessening the burden put on their children. Patients with AD were more likely to receive early LLST, earlier de-escalation of intensity of care which is converted into a shorter NCCU LOS. However, in patients receiving LLST, neither early LLST nor the presence of an AD were associated with a higher in-hospital mortality. The implementation of an AD might have, in these specific cases, limited unwanted and potentially not beneficial treatments, and improved quality of life.

We found no difference by sex in the reason for the LLST: this decision was equally based on a medical indication or the patients’ documented/presumed will. In Switzerland EOL discussions are carried out based on the principles of shared decision-making. The law protects the patients [25] and requires the treating physician as well as the SDM to comply with the patients’ known or presumed will. The presumed will can either be extracted from the AD (if available) or sought after from the SDM (based on the patients’ presumed will). The primary task of the physician is to inform patients/SDM and answer their questions regarding the outcome. The Swiss law allows both withholding as well as withdrawing LST in patients where the prolongation of survival would lead to an inacceptable quality of life. Patients in need of neurocritical care commonly lose the capacity to decide, thus an AD and an assigned SDM with knowledge of the patients’ wishes is of pivotal importance. Fitting prior reports, most caregivers in our cohort were women [13]. Women, when appointed as SDM, are less likely to seek formal support despite reporting higher levels of stress and often being affected by depression [26].

Our results reveal males as an important target for the education on AD, and a significant cultural gap when considering the overwhelming majority of patients with an AD being German speaking and of Swiss nationality. Sprung et al. [27] found no differences in LLST if the patients were Catholic or Protestant or without religious affiliation, while Jewish, Greek Orthodox, or Muslim patients were more likely to have their therapy withheld instead of withdrawn. This result could not be confirmed in our cohort. However, representative of the Swiss population [23], most of our patients were Catholic/Protestant or agnostic with other religions being underrepresented.

### Strengths and limitations

The strengths of this study lie in the large cohort assessing close to 3000 patients and the detailed description of social, cultural, and demographic factors and their association to LLST. Our results and conclusions are limited by the following factors: 1. The study’s single center design. 2. Detailed data was only available for patients that received LLST, thus comparison of associations was limited to these patients. 3. In-depth information regarding the SDM such as age, closeness of relationship as well as potential prior discussions regarding AD or EOL were unavailable. 4. While the CCI covers many important comorbidities, it is not exhaustive. Some pre-existing unnoted comorbidities might have influenced both LLST as well as the presence of an AD. 5. The decision-making process is left to the discretion of the attending intensivist and is thus susceptible to unconscious cognitive biases [28].

## Conclusions

The prevailing ethical principle in Switzerland is upholding patients’ autonomy: individuals have the right to make decisions based on their personal values and concepts. ADs are legally binding. Patients with AD were older, more likely female, and more likely received early LLST. However, the presence of an AD was not associated with an increased in-hospital mortality, which supports the notion that following the patients’ presumed will, will no per se lead to an unfavorable outcome.

## Data Availability

The datasets used and/or analyzed during the current study are available from the corresponding author on reasonable request.

## Abbreviations

aSAH: Aneurysmal subarachnoid hemorrhage
CCI: Charlson Comorbidity Index
CI: Confidence interval
EOL: End of life
GCS: Glasgow Coma Scale
ICH: Intracerebral hemorrhage
ICU: Intensive Care Unit
IQR: Inter Quartile Range
KESB: Kinder und Erwachsenen Schutzbehörde (child and adult protection authority)
LST: Life sustaining Treatments
LLST: Limitation of Life Sustaining Treatments
NCCU: Neuro Critical Care Unit
SDM: Surrogate decision maker
SOFA: Sequential organ failure assessment score
SAPS II: Simplified Acute Physiology Score II
